# Autoradiographical assessment of inflammation-targeting radioligands for atherosclerosis imaging: potential for plaque phenotype identification

**DOI:** 10.1186/s13550-021-00772-z

**Published:** 2021-03-17

**Authors:** Eric J. Meester, Erik de Blois, Boudewijn J. Krenning, Antonius F. W. van der Steen, Jeff P. Norenberg, Kim van Gaalen, Monique R. Bernsen, Marion de Jong, Kim van der Heiden

**Affiliations:** 1 Department of Biomedical Engineering, Thorax Center, Erasmus Medical Center, PO Box 2040, 3000 CA Rotterdam, The Netherlands; 2 Department of Radiology and Nuclear Medicine, Erasmus MC, Rotterdam, The Netherlands; 3 Department of Cardiology, Thorax Center, Erasmus MC, Rotterdam, The Netherlands; 4 Radiopharmaceutical Sciences, University of New Mexico, Albuquerque, NM USA

**Keywords:** Atherosclerosis, Inflammation, Molecular imaging, Autoradiography

## Abstract

**Purpose:**

Many radioligands have been developed for the visualization of atherosclerosis by targeting inflammation. However, interpretation of in vivo signals is often limited to plaque identification. We evaluated binding of some promising radioligands in an in vitro approach in atherosclerotic plaques with different phenotypes.

**Methods:**

Tissue sections of carotid endarterectomy tissue were characterized as early plaque, fibro-calcific plaque, or phenotypically vulnerable plaque. In vitro binding assays for the radioligands [^111^In]In-DOTATATE; [^111^In]In-DOTA-JR11; [^67^Ga]Ga-Pentixafor; [^111^In]In-DANBIRT; and [^111^In]In-EC0800 were conducted, the expression of the radioligand targets was assessed via immunohistochemistry. Radioligand binding and expression of radioligand targets was investigated and compared.

**Results:**

In sections characterized as vulnerable plaque, binding was highest for [^111^In]In-EC0800; followed by [^111^In]In-DANBIRT; [^67^Ga]Ga-Pentixafor; [^111^In]In-DOTA-JR11; and [^111^In]In-DOTATATE (0.064 ± 0.036; 0.052 ± 0.029; 0.011 ± 0.003; 0.0066 ± 0.0021; 0.00064 ± 0.00014 %Added activity/mm^2^, respectively). Binding of [^111^In]In-DANBIRT and [^111^In]In-EC0800 was highest across plaque phenotypes, binding of [^111^In]In-DOTA-JR11 and [^67^Ga]Ga-Pentixafor differed most between plaque phenotypes. Binding of [^111^In]In-DOTATATE was the lowest across plaque phenotypes. The areas positive for cells expressing the radioligand’s target differed between plaque phenotypes for all targets, with lowest percentage area of expression in early plaque sections and highest in phenotypically vulnerable plaque sections.

**Conclusions:**

Radioligands targeting inflammatory cell markers showed different levels of binding in atherosclerotic plaques and among plaque phenotypes. Different radioligands might be used for plaque detection and discerning early from vulnerable plaque. [^111^In]In-EC0800 and [^111^In]In-DANBIRT appear most suitable for plaque detection, while [^67^Ga]Ga-Pentixafor and [^111^In]In-DOTA-JR11 might be best suited for differentiation between plaque phenotypes.

**Supplementary Information:**

The online version contains supplementary material available at 10.1186/s13550-021-00772-z.

## Introduction

Inflammation plays a crucial role in atherosclerotic plaque formation, progression, and destabilization [1, 2]. Therefore, inflammation is an attractive imaging target for plaque detection [3]. Different plaque compositions present a different likelihood of rupture and subsequent cardiovascular events like myocardial infarction or stroke [4, 5]. Hence, there is a need for (A) plaque detection, and (B) a distinction between plaques in risk of rupture and in need of treatment as opposed to plaques which do not require intervention. As inflammation is a major factor in plaque destabilization, the level of inflammation might be used to identify vulnerable, rupture-prone plaques.

2-deoxy-2-[^18^F]fluoro-d-glucose ([^18^F]FDG) positron emission tomography/computed tomography (PET/CT) is taken up by metabolically active macrophages in plaque and has been shown to detect plaque inflammation in vivo [6]. However, [^18^F]FDG lacks specificity for inflammatory cells, and high uptake in the myocardium complicates image interpretation limiting the use of [^18^F]FDG in the coronary arteries [6, 7]. Therefore, recent research has focussed on the evaluation of other inflammation targeting radioligands.

A number of radioligands have shown good results for plaque detection in recent literature [8–10]. Especially imaging of the somatostatin subtype receptor 2 (SST_2_) and chemokine CXC motif receptor type 4 (CXCR4) seem promising. Coronary plaques were successfully detected by targeting SST_2_ on activated macrophages with [^68^Ga]Ga-[DOTA, Tyr^3^]-octreotate ([^68^Ga]Ga-DOTATATE) [11]. DOTA-JR11 (DOTA-Cpac[D-Cys-Aph(Hor)-D-Aph(Cbm)-Lys-Thr-Cys]-D-Tyr-NH_2_) also targets SST_2_ but has a reported five times higher uptake in tumors than DOTATATE in oncological studies, and a more favorable biodistribution resulting in a higher target to background ratio (TBR) [12–15]. Recently, we have reported on the successful use of [^111^In]In-DOTA-JR11 for plaque detection in atherosclerotic mice [16]. CXCR4 can be targeted with Pentixafor, which has shown favorable results for plaque visualization in a number of studies [17–21]. We found that targeting leukocyte function-associated antigen-1 (LFA-1) with radiolabelled DOTA-butylamino-NorBIRT (DANBIRT) was well suited for plaque detection [22, 23]. Moreover, we recently found higher uptake of radiolabelled DANBIRT than of an SST_2_-targeting radioligand ex vivo in human plaque tissue, as well as different levels of uptake in different plaque phenotypes [24]. Another promising radioligand is radiolabelled DOTA-Bz-EDA-folate (EC0800), which binds to the Folate Receptor (FR). We and others showed the feasibility of FR imaging for plaque detection with a number of different radioligands [25–27].

Previous studies mostly focused on plaque detection, and radioligand uptake is usually correlated to plaque presence, symptomatic plaque, or culprit plaque in the event of, e.g., myocardial infarction. To our knowledge, few studies relate radioligand uptake to plaque phenotype or composition (e.g., [28–30]). Ideally, future nuclear imaging methods relate radioligand uptake to plaque phenotype, ultimately identifying plaques requiring intervention before a major adverse cardiovascular event occurs. Therefore, we investigated radioligand binding in human plaque samples with different phenotypes. The aim of this study was to investigate which radioligands have a high potential to detect plaque, and which radioligands are most suited to differentiate between different plaque phenotypes. As PET radionuclides offer poor resolution for in vitro binding assays, we labelled the radioligands with SPECT radionuclides.

## Methods

### Study material

Human carotid plaques were obtained with informed consent via carotid endarterectomy from eight patients in the Erasmus MC. Sample acquisition was approved by the medical ethics committee of the Erasmus MC (MEC 2008-147). The samples were snap-frozen in liquid nitrogen and stored in − 80 °C until the experiment started to allow minimal variation between experiments due to, e.g., variations in labelling conditions. Freezing and thawing of tissue is common method of tissue storage, yet might degrade tissue quality [31–33]. The samples were embedded in Tissue-Tek O.C.T. compound (Sakura Finetek Europe B.V, Alpen aan den Rijn, The Netherlands) and stored in − 80 °C, after which tissue sections for in vitro binding assays and immunohistochemistry were sectioned at 4 mm intervals. All sections were included in the study (*n* = 37). The amount of required samples was based on an earlier study performed in our group [24].

### Tissue sectioning

Tissue was sectioned at 5 µm for immunohistochemical (IHC) analysis and at 10 µm for in vitro binding assays [autoradiography (ARG)]. In vitro binding assays were performed on adjacent sections for optimal comparison. Similarly, IHC was performed on sections adjacent to the sections used for in vitro binding assays.

### Radiolabelling

DOTATATE, DOTA-JR11 (provided by Helmut Maecke), DANBIRT, and EC0800 (provided by Auspep, Tullamarine, Australia) were labelled with [^111^In]InCl_3_ (Covidien, Petten, The Netherlands) with a molar activity of 200 MBq/nmol as described previously [34]. Pentixafor (provided by Hans-Jürgen Wester) was labelled with Gallium-67 (Curium, Petten, The Netherlands), as Indium-111 labelling results in reduced binding affinity [35, 36]. Although Pentixafor is regularly labelled with Ga-68, we used Ga-67 due to its preferable characteristics for autoradiography in terms of half-life and resolution. Labelling with Gallium-67 was performed with a molar activity of 100 MBq/nmol as described [37]. Radiochemical purity (> 95%) and incorporation yield (> 99%) were evaluated with high-pressure liquid chromatography and instant thin-layer chromatography on silica gel. Quenchers were added to prevent radiolysis as described previously [38, 39].

### In vitro binding assays and competition binding assays

Sections were incubated for 1 h with 80 µL 10^–9^ M radiolabelled ligand, the incubation time was determined in our institute and the same for all investigated radioligands. Specificity of the radioligands was demonstrated previously [11, 16, 17, 40–44]. Additionally, we confirmed specific binding in our tissue via competition binding experiments by blocking with 10^–6^ M unlabelled compound (see Additional file 1: Table S1 in the data supplement). Standards (1 µL of 1:10; 1:100; and 1:1000 diluted incubation buffer in triplo) were added to normalize data. Slides were exposed to phosphor screens overnight and read with a phosphor imager (Cyclone, Perkin Elmer).

### Immunohistochemistry

Sections were immunohistochemically stained for SST_2_ (ab134152, Abcam), CXCR4 (ab124824, Abcam), LFA-1 (MCA1848, AbD SeroTec), or FR (AP5032a, Abgent). In short, sections were fixed in cold acetone for 5 min (SST_2_, CXCR4, LFA-1) or 10% formalin for 10 min (FR), endogenous peroxidase was blocked with 0.3% H_2_O_2_ for 30 min (SST_2_, LFA-1, FR) or 0.15% H_2_O_2_ for 20 min (CXCR4), and non-specific binding was blocked with 1% BSA for 20 min (CXCR4) or 2% normal goat serum for 20 min (SST_2_, LFA-1, FR). The primary antibody was omitted from the protocol in negative controls.

Haematoxylin–eosin staining according to standard protocol was performed on the sections used for in vitro binding assays after radioactivity had sufficiently decayed. These sections were later used for plaque classification.

### Plaque classification

Hematoxylin–eosin stained plaque sections where classified by two independent observers according to the criteria used in the adapted American Heart Association (AHA) classification [4, 5, 45]. Plaque phenotypes were categorized into three groups: early plaque; fibro-calcific (FCALC) as stable plaque; and phenotypically vulnerable plaque. Hematoxylin–eosin staining allows the visualization of plaque calcifications [46, 47] to classify FCALC phenotypes. Typical examples of each category are visible in Additional file 1: Figure S1 of the data supplement.

### Quantification and statistical analysis

Radioactive signal in whole plaque sections was quantified with Optiquant (Perkin Elmer) by delineating the tissue section based on histology, and expressed in digital light units (DLU). Signal was corrected for radionuclide half-life and exposure time of the phosphor screen to the tissue sections. The amount of DLUs in the standards was quantified via which the percentage of added activity per incubated section was calculated by dividing the DLUs per section by the total amount of DLUs in the 80 µL incubation medium. The percentage added activity was divided by the surface area of each section (in mm^2^) to calculate the percentage added activity per mm^2^ (%AA/mm^2^). For immunohistochemically-stained sections, Biopix software (Biopix AB, Gothenburg, Sweden) was used to calculate the percentage DAB (3,3′-diminobenzidine) positive area per tissue section.

The data were analyzed with SPSS 21.0 (SPSS Inc, Chicago, IL). The data was tested for normality using the Shapiro–Wilk test. Differences between groups were analyzed with the Kruskal–Wallis test, with Dunn’s post hoc test to account for multiple comparisons. *p* values below 0.05 were considered significant. Data are presented as mean ± standard deviation.

## Results

### Plaque classification

Plaque sections were classified as early plaque (12 samples), stable plaque (10 samples), or phenotypically vulnerable plaque (15 samples) with an interobserver agreement of 97%.

### Area of DAB positive staining and radioligand binding

#### Area of cells expressing radioligand targets

**Fig. 1:**
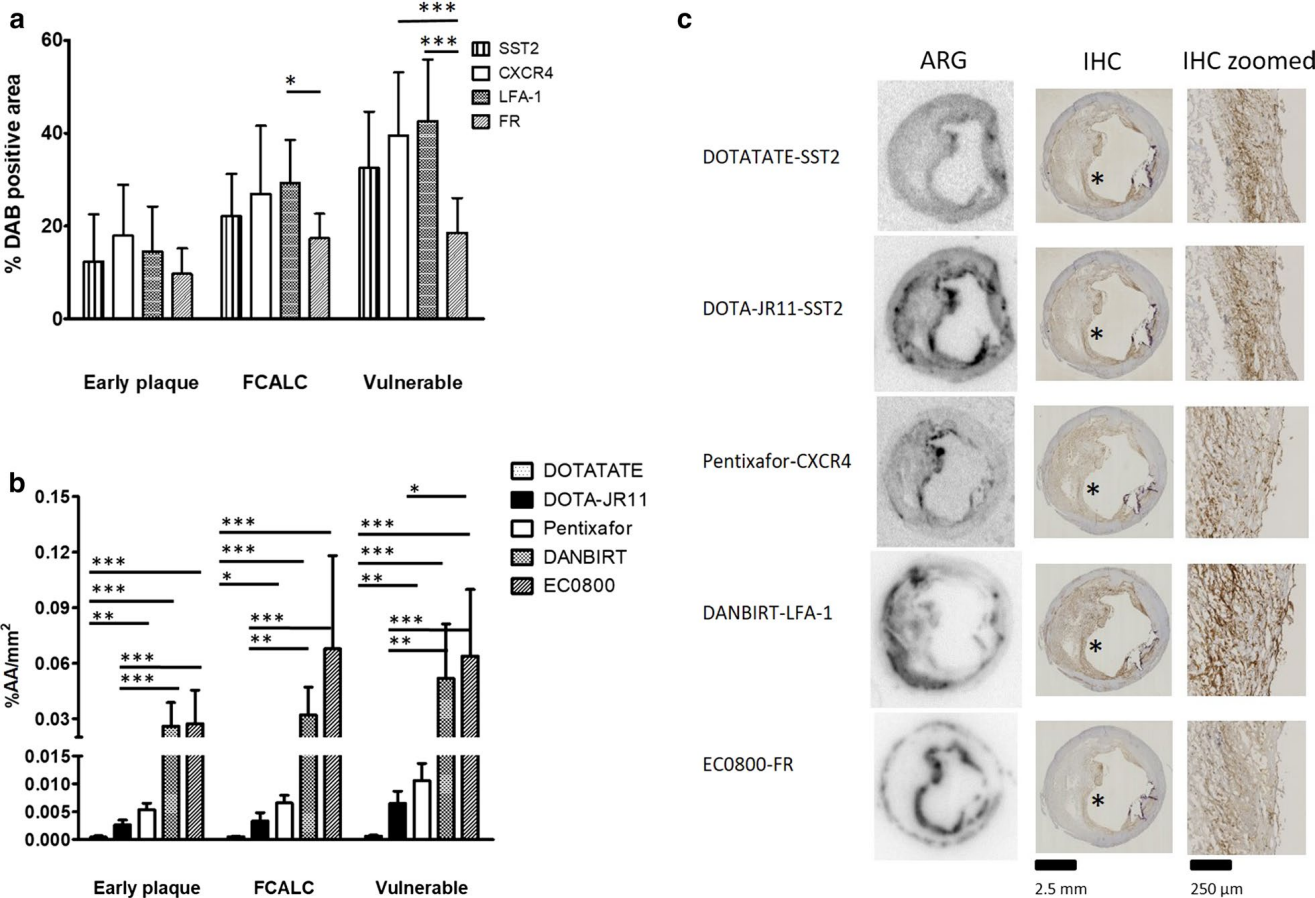
**a** % DAB positive area in sections categorized as early plaque, stable plaque (FCALC), and vulnerable plaque. **b** % Added activity/mm^2^ (%AA/mm^2^) per radioligand in different plaque categories. Note that the *y*-axis consists of two segments. **c** Representative examples of autoradiograms of the different radioligands and immunohistochemistry of adjacent sections stained for the radioligand target. The autoradiograms are not uniformly scaled, but scaled for visibility per image. DOTATATE, DOTA-JR11, DANBIRT, and EC0800 were labelled with Indium-111, Pentixafor was labelled with Gallium-67. Bars indicate mean with standard deviation. *indicates *p* < 0.05, **indicates *p* < 0.01, ***indicates *p* ≤ 0.001. *ARG* autoradiography, *IHC* immunohistochemistry, *SST*_*2*_ somatostatin subtype receptor 2, *CXCR4* chemokine CXC motif receptor type 4, *LFA-1* leukocyte associated antigen 1, *FR* folate receptor, *FCALC* fibro-calcific, *DAB* 3,3′-Diaminobenzidine

Figure 1a shows the average DAB-positive area for each studied radioligand target and plaque category. All radioligand targets were expressed in larger areas in more advanced plaques than in early plaques (Fig. 1a). No significant differences in areas were observed in early plaque sections. In FCALC plaque sections, the areas containing LFA-1 expressing cells were significantly higher than the areas of FR expressing cells (*p* < 0.05). In vulnerable plaque sections, FR was expressed in significantly less area than LFA-1 (*p* < 0.001) and CXCR4 (*p* < 0.001).

#### Binding of [^111^In]In-DANBIRT and [^111^In]In-EC0800 was highest across plaque phenotypes

Figure 1b shows radioligand binding of the different radioligands across plaque categories. Binding of [^111^In]In-DANBIRT and [^111^In]In-EC0800 was highest, followed by [^67^Ga]Ga-Pentixafor, [^111^In]In-DOTA-JR11, and [^111^In]In-DOTATATE. In sections of early plaque lesions, [^111^In]In-DOTATATE signal (0.00047 ± 0.00017%AA/mm^2^) was significantly lower than [^67^Ga]Ga-Pentixafor (0.0053 ± 0.0012%AA/mm^2^ *p* < 0.01), [^111^In]In-DANBIRT (0.026 ± 0.013%AA/mm^2^) and [^111^In]In-EC0800 (0.027 ± 0.018%AA/mm^2^) signal (*p* < 0.001). [^111^In]In-DOTA-JR11 signal (0.0027 ± 0.00085%AA/mm^2^) was also significantly lower than [^111^In]In-DANBIRT and [^111^In]In-EC0800 signals (*p* < 0.001).

In FCALC sections, [^111^In]In-DOTATATE signal (0.00053 ± 0.000076%AA/mm^2^) was also significantly lower compared to [^67^Ga]Ga-Pentixafor (0.0066 ± 0.0014%AA/mm^2^ *p* < 0.05), [^111^In]In-DANBIRT (0.032 ± 0.015%AA/mm^2^) and [^111^In]In-EC0800 (0.068 ± 0.050%AA/mm^2^) (*p* < 0.001). [^111^In]In-DOTA-JR11 signal (0.0033 ± 0.0015%AA/mm^2^) was also significantly lower than [^111^In]In-DANBIRT (*p* < 0.01) and [^111^In]In-EC0800 (*p* < 0.001).

In vulnerable plaque sections, [^111^In]In-DOTATATE signal (0.00064 ± 0.00014%AA/mm^2^) was again lower than that of [^67^Ga]Ga-Pentixafor (0.011 ± 0.003%AA/mm^2^ *p* < 0.05), [^111^In]In-DANBIRT (0.052 ± 0.029%AA/mm^2^) and [^111^In]In-EC0800 (0.064 ± 0.036%AA/mm^2^ *p* < 0.001). [^111^In]In-DOTA-JR11 signal (0.0066 ± 0.0021%AA/mm^2^) was significantly lower than those of [^111^In]In-DANBIRT (*p* < 0.01) and [^111^In]In-EC0800 (*p* < 0.001), whereas [^111^In]In-EC0800 signal was significantly higher than [^67^Ga]Ga-Pentixafor signal (*p* < 0.05).

#### Radioligand binding was higher in advanced plaque phenotypes than in early plaque

**Fig. 2:**
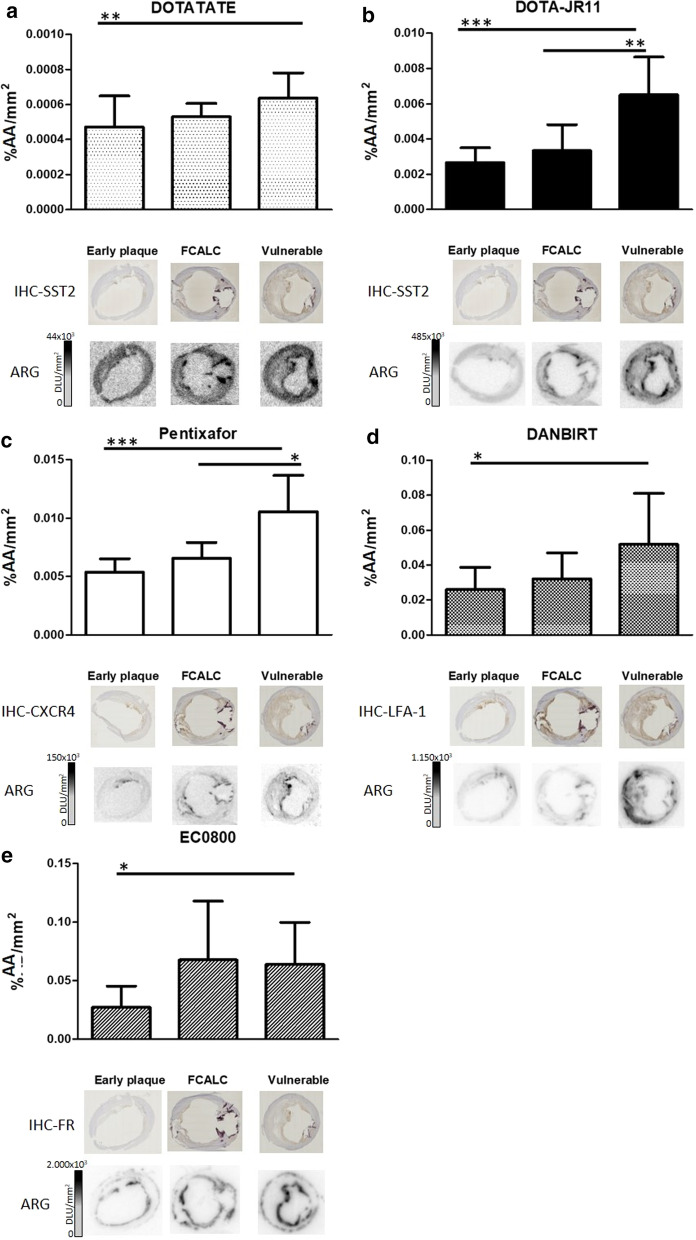
% added activity/mm^2^ (%AA/mm^2^) in plaque sections for each radioligand across plaque categories. Representative examples of IHC and ARG are shown below each radioligand graph. DOTATATE, DOTA-JR11, DANBIRT, and EC0800 were labelled with Indium-111, Pentixafor was labelled with Gallium-67. Bars indicate mean with standard deviation. *indicates *p* < 0.05, **indicates *p* < 0.01, ***indicates *p* ≤ 0.001. *ARG* autoradiography, *IHC* immunohistochemistry, *SST*_*2*_ somatostatin subtype receptor 2, *CXCR4* chemokine CXC motif receptor type 4, *LFA-1* leukocyte associated antigen 1, *FR* folate receptor

Figure 2 shows radioligand binding per radioligand across plaque phenotypes, Additional file 1: Figure S2 shows a more elaborate data presentation with individual data points. All radioligands showed more signal in advanced than in early plaque sections. [^111^In]In-DOTATATE signal was significantly higher in vulnerable sections than in sections classified as early plaque (0.00064 ± 0.00014 and 0.00047 ± 0.00017%AA/mm^2^ *p* < 0.01). [^111^In]In-DANBIRT and [^111^In]In-EC0800 also had significantly higher signal in vulnerable sections compared to early plaque (0.052 ± 0.029 vs 0.026 ± 0.013 and 0.064 ± 0.036 vs 0.027 ± 0.018%AA/mm^2^, respectively. *p* < 0.05). [^111^In]In-DOTA-JR11 bound significantly more to vulnerable sections compared to early (0.0066 ± 0.0021 vs 0.0027 ± 0.00085%AA/mm^2^ *p* < 0.001) and FCALC sections (0.0033 ± 0.0015%AA/mm^2^ *p* < 0.01). [^67^Ga]Ga-Pentixafor showed a similar binding pattern as [^111^In]In-DOTA-JR11, with binding in vulnerable sections being higher (0.011 ± 0.003%AA/mm^2^) than binding in early plaque sections (0.0053 ± 0.0012%AA/mm^2^ *p* < 0.001) and FCALC sections (0.0066 ± 0.0014%AA/mm^2^ *p* < 0.05).

#### [^111^In]In-DOTA-JR11 binding is higher than [^111^In]In-DOTATATE

There was a clear difference in binding amongst the SST_2_ targeting radioligands, but the differences were not statistically significant. [^111^In]In-DOTA-JR11 showed a 5.6 fold higher signal than [^111^In]In-DOTATATE in early plaque sections, a 6.3 fold higher signal in stable plaques, and a 10.2 fold higher signal in vulnerable plaque sections (Fig. 1b).

#### Location of binding differs between radioligands

Figure 1c illustrates the differences in radioligand signal and target receptor distribution in vulnerable plaque sections. Figure 2 displays representative examples of radioligand binding and target receptor distribution. Expression of SST_2_ and signal of [^111^In]In-DOTATATE and [^111^In]In-DOTA-JR11 was mostly located in cap areas of plaque. [^67^Ga]Ga-Pentixafor signal and CXCR4 expression was located in cap areas as well as around calcified nodes. [^111^In]In-DANBIRT signal and LFA-1 expression was visible on the medial side of the necrotic core, in cap areas, and around calcium deposits. Signal of [^111^In]In-EC0800 and expression of FR was mostly located at areas close to the artery lumen, and in areas at the edge of the tunica media. Signal of [^67^Ga]Ga-Pentixafor and [^111^In]In-DOTA-JR11 differed most between vulnerable plaque sections and early or stable plaque sections.

## Discussion

A large number of inflammation targeted radioligands has been studied for atherosclerotic plaque detection [8–10]. We examined the binding of several promising inflammation targeted radioligands and the distribution of cells expressing the radioligand targets in sections of different plaque phenotypes. For all radioligands, we found higher levels of binding in advanced, vulnerable plaque sections than in early plaque sections. The same pattern was visible for the portion of plaque area expressing the radioligand targets. Our key findings highlight how in vitro binding differs between these radioligands and between plaque phenotypes. The results indicate that some radioligands might be better suited for plaque detection, with a higher uptake across plaque phenotypes, whereas other radioligands appear useful to differentiate between plaque phenotypes and therefore identify plaques in need of treatment. Specifically, we found significantly higher binding of [^111^In]In-DANBIRT and [^111^In]In-EC0800 across plaque phenotypes compared to the other radioligands. Binding of [^111^In]In-DOTA-JR11 and [^67^Ga]Ga-Pentixafor differed the most between vulnerable plaque sections and sections of the other plaque phenotypes. These results could help interpret results of in vivo studies, expanding meaning of uptake in nuclear scans beyond plaque presence or identification of culprit plaque.

We studied in vitro binding of radioligands in tissue sections of atherosclerotic plaque. Binding differs strongly between in vitro assays and in vivo. The mechanisms of uptake could differ, expression of receptors might be influenced differently in vivo, and radioligands in vivo often only get a limited number of passes along tissues to reach their target receptor before being cleared from the blood. Moreover, background signal from other tissues might be high in vivo. Also, the effects of freezing or tissue degradation can play a role in in vitro binding. Despite these important differences between in vitro and in vivo, our approach provides valuable information. The targets are optimally reachable in 10 µm sections, the radioligands get sufficient time to bind to available receptors, and there are no imaging artefacts related to resolution, spill over, or attenuation. Therefore, our approach mimics the potential binding of the radioligands in the examined tissues and might give an indication of an idealized uptake that might be achieved in vivo.

We examined radioligand binding using SPECT radionuclides, as these offer a better spatial resolution in autoradiography than PET radionuclides. However, in a clinical setting PET is preferred as it has a higher spatial resolution than SPECT. Although labelling with different radionuclides can result in differences in binding affinity, all investigated targets have been examined with PET radionuclides with good results. We therefore believe our results are translatable to clinical practice.

Recent studies suggest that SST_2_ targeting with DOTATATE is a viable strategy for plaque detection [40, 48–52]. Tarkin and colleagues showed that [^68^Ga]Ga-DOTATATE could better discriminate between high-risk and low-risk plaques compared to [^18^F]FDG [11]. However, another study found that imaging with [^68^Ga]Ga-DOTATATE could not differentiate between symptomatic plaque and the contralateral artery, and in line with these results found no SST_2_ expressing cells in plaque [53]. Furthermore, the aorta to blood ratio of [^68^Ga]Ga-DOTATATE found by Rinne et al*.* [49] was lower than one, which further indicates that in vivo imaging with this radioligand requires optimization. Our in vitro results could in part explain these discrepancies. We clearly demonstrated the presence of SST_2_ expressing cells in human carotid tissues and found localized binding of [^111^In]In-DOTATATE. However, [^111^In]In-DOTATATE signal was much lower than signal of the other studied radioligands. Moreover, binding of [^111^In]In-DOTATATE between sections of different plaque phenotypes showed the least difference of all investigated radioligands. Although imaging of atherosclerosis with DOTATATE is valid for plaque detection, these results confirm the need for further optimization.

DOTA-JR11 could provide a significant improvement in SST_2_ imaging. Oncological studies reported a much higher uptake and TBR of DOTA-JR11 than DOTATATE, ranging from 2 to 20 fold more [12–15]. The mechanism for this difference in uptake between the SST_2_ targeting radioligands remains to be elucidated, it is hypothesized that antagonistic ligands such as DOTA-JR11 bind more binding sites than agonistic ligands such as DOTATATE [54]. We recently found that [^111^In]In-DOTA-JR11 could be used to detect atherosclerotic plaques in vivo in an animal model of atherosclerosis with high specificity [16]. Our current results showed a 10.2 fold higher signal of [^111^In]In-DOTA-JR11 over [^111^In]In-DOTATATE in vulnerable plaque sections. Moreover, binding of [^111^In]In-DOTA-JR11 differed significantly between plaque phenotypes, which indicates that DOTA-JR11 could be useful to discriminate between different plaque phenotypes. These results strongly suggest that imaging SST_2_ with DOTA-JR11 is a relevant strategy for plaque detection and characterization.

Similarly to DOTA-JR11, we found high binding of [Ga^67^]Ga-Pentixafor, and significantly different binding between plaque phenotypes. In line with our findings, Derlin et al. [18] found more CXCR4 expressing cells in symptomatic plaques than in asymptomatic plaque tissue. Similar findings were reported via mRNA and IHC assays, with more CXCR4 expression in late stage plaque [21, 55, 56]. Combined with our data these and other studies mark Pentixafor as an important radioligand for atherosclerosis detection and characterization [17, 19, 20, 57–59].

[^111^In]In-DANBIRT binding differed strongly from binding of [^111^In]In-DOTATATE, [^111^In]In-DOTA-JR11, and [Ga^67^]Ga-Pentixafor. Binding of [^111^In]In-DANBIRT was located at the same locations, but was in addition high at the medial side of the necrotic core. The high signal and binding in more areas compared to the other radioligands was in line with the target findings. Earlier studies showed high specificity of DANBIRT, and in vivo experiments in animal models of atherosclerosis show promising results [23, 42, 43]. Moreover, our group also found different levels of [^111^In]In-DANBIRT uptake in phenotypically different plaque tissues ex vivo [24]. Combined with the high binding of [^111^In]In-DANBIRT in our tissue sections this suggests that this radioligand is relevant for plaque detection and characterization.

[^111^In]In-EC0800 binding was highest across plaque phenotypes in the examined panel of radioligands, and its signal was significantly higher in sections classified as vulnerable plaque than in early plaque sections. Imaging of FR is extensively studied in atherosclerosis with different imaging probes in vivo in animal models of atherosclerosis [26, 27, 60, 61] and in vitro in human tissue [27, 62–64]. Müller et al*.* found higher FR expression and higher signal of a FR targeting radioligand in sections of plaque compared to normal arterial wall, but no significant differences between sections of plaque classified as stable or vulnerable [64]. In contrast, our group showed in vivo uptake of [^111^In]In-EC0800 in a mouse model of atherosclerosis and found higher [^111^In]In-EC0800 uptake in stable plaque compared to vulnerable plaque [26]. Jager et al*.* linked FR expression to presence of M1-like macrophages [62]. None of the studies examining in vitro expression or imaging of FR reported uptake or binding in the edges of tissue sections, like we observed in our study. FR staining confirmed FR expression in these locations, therefore binding of EC0800 in these locations is not an artefact. However, additional staining for CD68 (data not shown) shows no presence of macrophages in these areas. FR expression is not limited to macrophages, further studies into the binding or uptake of FR targeting radioligands are therefore recommended.

Few imaging studies make head-to-head comparisons between radioligands or relate radioligand uptake to plaque composition beyond identification of clinically identified culprit plaque. Our and various other studies can aid the development of new imaging strategies [29, 49] and are useful to translate in vivo signal to characterization of plaque status. For example, Rinne et al*.* examined multiple radioligands in an in vivo model of atherosclerosis, and Borchert et al*.* investigated uptake of multiple radioligands in vitro in different leukocyte subtypes [29, 49]. Such studies can be used to identify which radioligands are most suited for plaque detection and for identification of vulnerable plaques in need of intervention. Our results warrant further clinical translation studies of plaque imaging using these new radioligands.

## Conclusion

This in vitro study indicates that DANBIRT and EC0800 are most suited for plaque detection given the high levels of binding. DOTA-JR11 and Pentixafor are best suited to differentiate between stable and vulnerable plaque based on the high differences in binding of these radioligands in phenotypically stable and vulnerable plaques. As in vivo studies with these radioligands show promising results for plaque detection, our findings indicate that further clinical evaluations for plaque characterization are warranted.

## Supplementary Information

**Additional file 1: Supplemental Figure 1**. Haematoxylin-eosin stained plaque sections of typical examples of early, FCALC, and vulnerable plaque. **Supplemental Figure 2**. Expression of radioland targets and binding of radioligands in plaque sections. **Supplemental Table 1**. Results of blocking studies.
