# Supplementary material for: Autoradiographical assessment of inflammation-targeting radioligands for atherosclerosis imaging: potential for plaque phenotype identification
Source: EJNMMI Res. 2021 Mar 17;11:27. doi: 10.1186/s13550-021-00772-z (PMC7969682; doi:10.1186/s13550-021-00772-z)
Supplement: Supplementary file 1 — Additional file 1: Supplemental Figure 1. Haematoxylin-eosin stained plaque sections of typical examples of early, FCALC, and vulnerable plaque. Supplemental Figure 2. Expression of radioland targets and binding of radioligands in plaque sections. Supplemental Table 1. Results of blocking studies. [file 13550_2021_772_MOESM1_ESM.docx]

## Data supplement


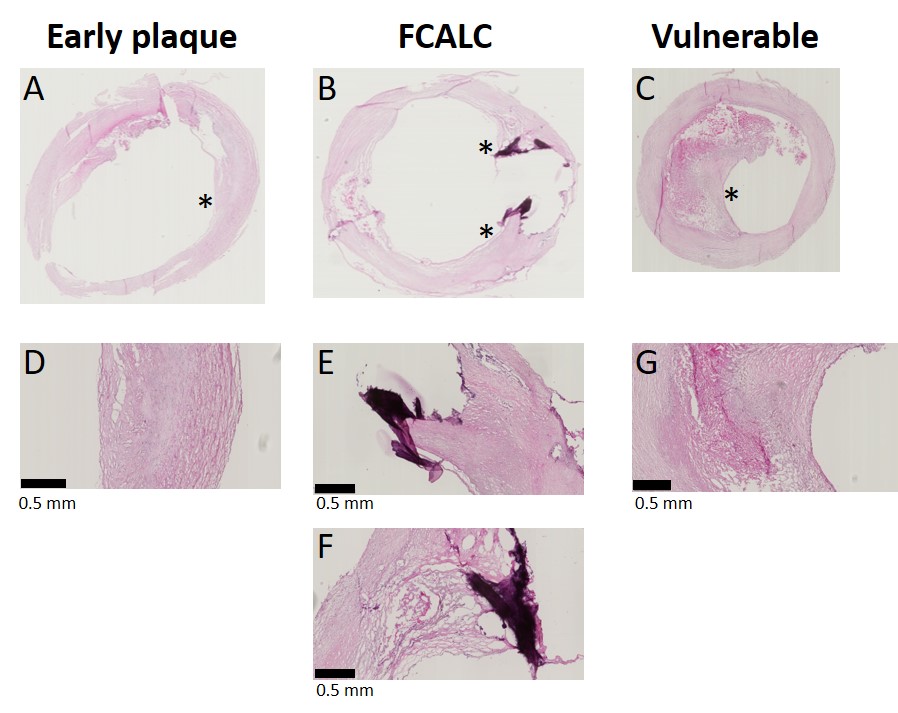


Supplemental Figure 1: Haematoxylin-eosin stained plaque sections of typical examples of A) early plaque; B) fibro-calcific (FCALC) as stable plaque; and C) phenotypically vulnerable plaque. Asterisks indicate the location of the magnifications in D-G. D) Magnification of A, in which lipid accumulations without a clear fibrous cap or necrotic core are visible. E) Shows a magnification of B, in which a collagen rich fibrous cap is visible, as well as a location where a large calcification has been pushed out of the section during sectioning. F) Magnification of B, in which a small area of necrosis is visible. G) Shows a magnification of C, where a thin cap with an underlying necrotic core is visible.


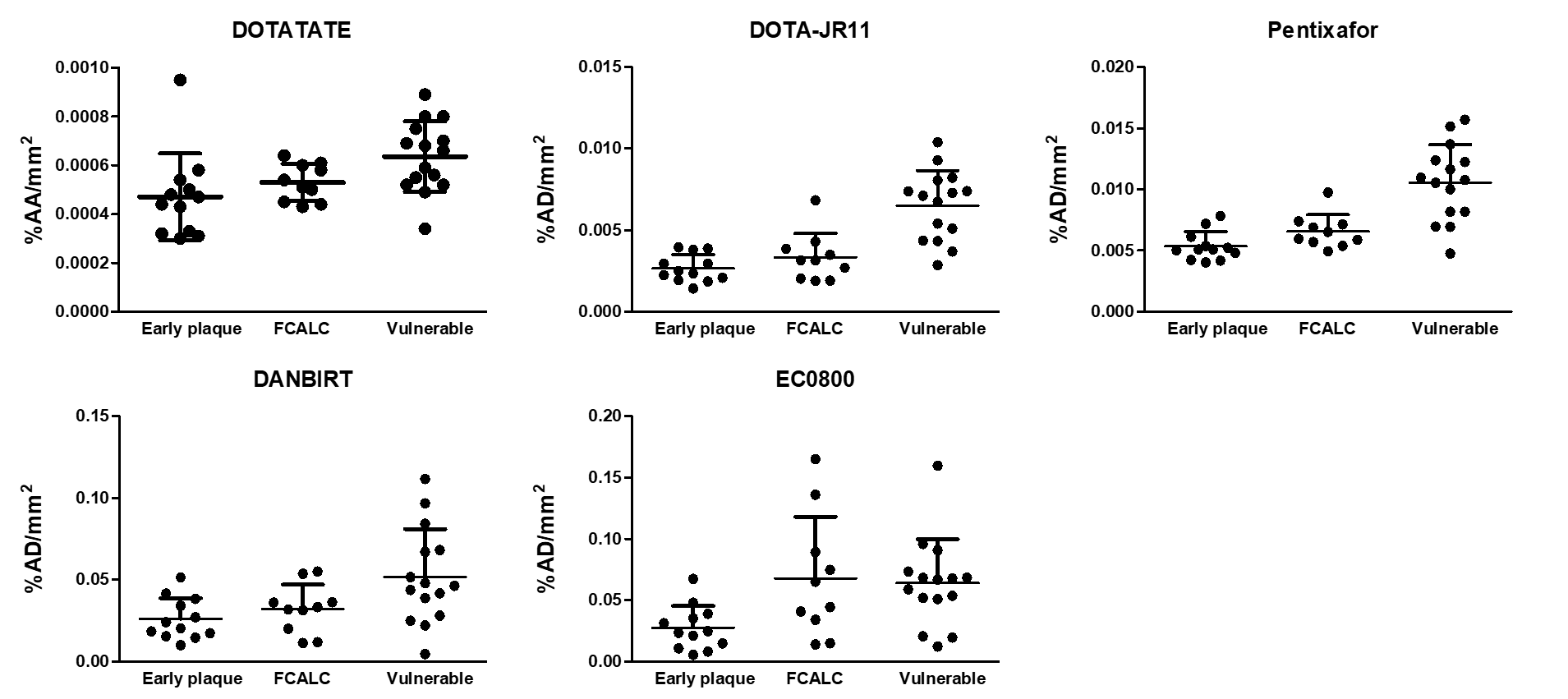


Supplemental Figure 2: Displays the individual data points depicted in Figure 2 for the % added activity/mm^2^ (%AA/mm^2^) in plaque sections for each radioligand across plaque categories. Note that the graphs are not uniformly scaled. DOTATATE, DOTA-JR11, DANBIRT, and EC0800 were labelled with Indium-111, Pentixafor was labelled with Gallium-67. Bars indicate mean with standard deviation.

Supplemental Table 1: Digital light units/mm^2^ (DLU/mm^2^) for blocked sections (incubated with 10^-9^ M labelled compound+10^-6^ M unlabelled compound) versus unblocked sections (incubated with 10^-9^ M labelled compound), ± standard deviation. Data were tested for normality, and paired t-test or Wilcoxon signed rank test was used to test if blocking significantly reduced binding.

| aVERAGE DLU/mm^2^ IN BLOCKED AND NON-BLOCKED SECTIONS | | |  |
| --- | --- | --- | --- |
|  | Average non blocked | Average blocked | Difference significant? |
| DOTATATE | 58 x10^3^ ± 6.6 x10^3^ | 44.9 x10^3^ ± 3.5 x10^3^ | *** |
| JR11 | 306.6 x10^3^ ± 44.8 x10^3^ | 196.6 x10^3^ ± 30.6 x10^3^ | ** |
| Pentixafor | 155.1 x10^3^ ± 22.9 x10^3^ | 80.5 x10^3^ ± 19.9 x10^3^ | ** |
| DANBIRT | 1116 x10^3^ ± 259.8 x10^3^ | 463.1 x10^3^ ± 53.3 x10^3^ | * |
| EC0800 | 2143.1 x10^3^ ± 380.6 x10^3^ | 58.7 x10^3^ ± 11.1 x10^3^ | ** |
